# Recurrent breast abscess caused by *Lawsonella clevelandensis*: A case report and literature review

**DOI:** 10.1097/MD.0000000000044218

**Published:** 2025-08-29

**Authors:** Yuzhen Liu, Xu Yue, Yujun Zhou, Junling Wang, Fang Yang, Yanling Jin, Guohui Zhang

**Affiliations:** a Department of Ultrasound, Kunming Medical University Affiliated Qujing Hospital, Qujing, China; b Department of Vascular Surgery, Kunming Medical University Affiliated Qujing Hospital, Qujing, China; c Department of Infection, Kunming Medical University Affiliated Qujing Hospital, Qujing, China; d Clinical Laboratory, Kunming Medical University Affiliated Qujing Hospital, Qujing, China; e Department of Hematology, Kunming Medical University Affiliated Qujing Hospital, Qujing, China.

**Keywords:** breast abscess, infections, *Lawsonella clevelandensis*, pathogens

## Abstract

**Rationale:**

*Lawsonella clevelandensis* is a gram-positive bacterium, partially acid-fast, strictly anaerobic, nonspore-forming, and catalase-positive. This microorganism was once overlooked in clinical microbiology due to its stringent growth requirements in laboratory cultures, but it has recently attracted recognition as a potential pathogen. Available reports implicate *Lawsonella clevelandensis* infection with abscess formation, including breast, spinal, abdominal, and deep soft tissue abscesses. Here, we present a case of recurrent breast abscess caused by *Lawsonella clevelandensis* infection.

**Patient concerns:**

A 46-year-old female patient had a history of left breast abscess for 6 years, with recurrent episodes and persistent symptoms.

**Diagnoses:**

Three milliliters of pus obtained from abscess puncture were examined. The results were negative for tuberculosis, interferon-γ, the rifampicin resistance gene *rpoB*, and *Mycobacterium tuberculosis* complex. The Kingfield metagenomics capture (MetaCAP) test identified *Lawsonella clevelandensis* (sequence number 17,296) with a 99% confidence level and no detected resistance genes.

**Interventions:**

Following abscess puncture and irrigation under ultrasound guidance, intravenous infusion of “piperacillin-tazobactam (4.5 g q8h)” was administered for 16 days, resulting in an improvement in the patient’s condition. Oral treatment with “amoxicillin-clavulanate 2.0 g bid and metronidazole 1.2 g tid” was continued after discharge.

**Outcomes:**

One month after discharge, color Doppler ultrasound showed a significant reduction in the abscess size. At the 3-month telephone follow-up, the patient reported that she had not taken the medication for over a month and had experienced no symptoms of redness, swelling, or pain in the left breast.

**Lessons:**

The clinical manifestations of *Lawsonella clevelandensis* infection are similar to those of other acid-fast bacilli (e.g., *Nocardia* and *Mycobacterium tuberculosis*), potentially leading to misdiagnosis and mistreatment, thereby delaying resolution of the condition. The methods used for treating infections by bacterial pathogens differ significantly, as do the prognoses, indicating the importance of precise diagnosis. *Lawsonella clevelandensis* should be included in the differential diagnosis of infections caused by acid-fast bacteria. Due to the extreme difficulty in culturing this bacterium in vitro, gene sequencing is used primarily for diagnosis. Overall, the prognosis of patients with *Lawsonella clevelandensis* infection is good. Timely debridement and drainage, combined with antibiotic treatment, can usually lead to a cure.

## 1. Introduction

Breast abscesses are a form of inflammatory lesions in the breast, which are usually caused by untimely or incomplete treatment of acute mastitis. While the disease is common in lactating women, it can also occur in other age groups. The most common pathogenic bacteria are *Staphylococcus aureus* and *Streptococcus*. This report describes a case of breast abscess caused by infection with the uncommon bacterial pathogen *Lawsonella clevelandensis* and reviews the relevant literature.

## 2. Case presentation

A 46-year-old female presented with a history of severe, stabbing pain in the upper quadrant of her left breast, which began spontaneously 6 years ago. The symptoms progressively worsened, characterized by local skin redness, swelling, ulceration, and pus discharge. Initial anti-infection treatments were ineffective. Five years ago, she underwent incision and drainage of the abscess at our hospital, where a pus smear revealed acid-fast bacilli (+1). Given the suspicion of a tuberculous infection of the left breast, she responded positively to a diagnostic regimen of quadruple antituberculosis therapy. After adhering to the medication regimen for 3 months, she discontinued the prescribed course on her own and self-medicated with isoniazid (0.3 g daily) for nearly 5 years. During this period, she experienced recurrent episodes of redness and swelling in the affected breast, with temporary relief following intermittent intravenous penicillin infusions.

**Figure 1.**
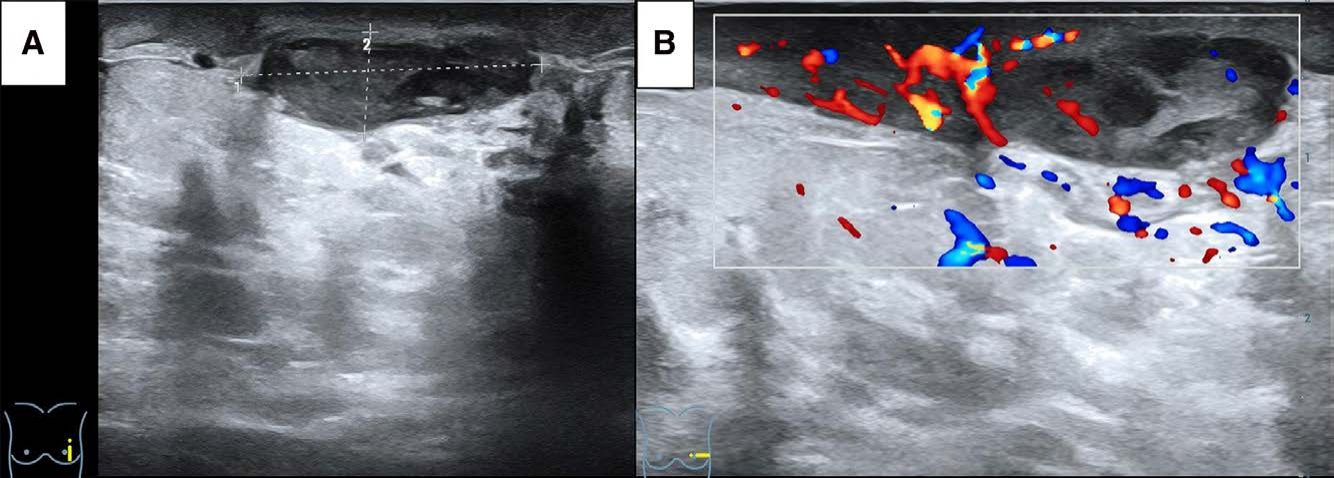
Parapillary abscess located at the 2 to 3 o’clock position on the left breast. (A) Longitudinal section measuring approximately 28 mm by 10 mm. (B) Transverse CDFI demonstrating significant blood flow surrounding the abscess. CDFI = color Doppler flow imaging.

**Figure 2.**
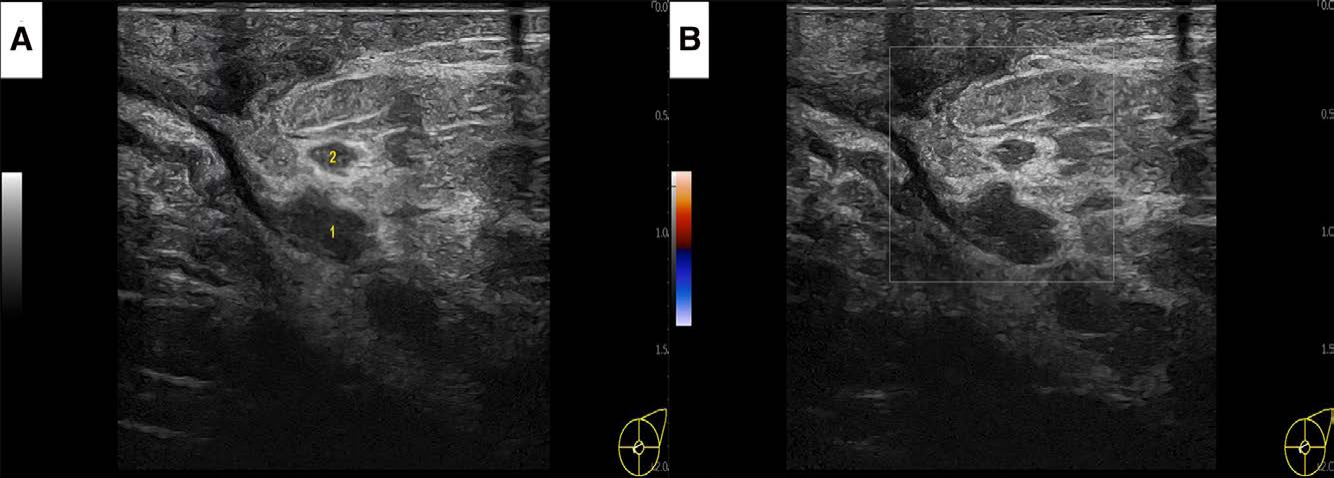
Parapillary abscess located at the 2 to 3 o’clock position on the left breast. (A) The abscess has diminished in size, appearing as multiple low-echo areas, with 1 patch measuring approximately 10 mm by 7 mm; (B) CDFI showed no significant blood flow within the lesion. CDFI = color Doppler flow imaging.

Her condition deteriorated recently, prompting a return visit to our hospital on June 16, 2024. Physical examination shows local skin redness, swelling, tenderness, nipple pus discharge. A breast ultrasound was performed (Fig. 1), and approximately 3 mL of pus was aspirated under ultrasound guidance for further testing. The results were negative for tuberculosis interferon-γ, the rifampicin resistance gene rpoB, and Mycobacterium tuberculosis complex. However, the Kingfield metagenomics capture (MetaCAP) test identified *Lawsonella clevelandensis* (sequence number 17,296) with a 99% confidence level (Table 1) and no detected resistance genes, indicating an infection with this bacterium. The initial selection of piperacillin-tazobactam was empirically guided by its broad-spectrum activity, including anaerobic coverage, to account for the possibility of polymicrobial infection commonly seen in chronic breast abscesses. This approach was also informed by literature reports suggesting *Lawsonella clevelandensis* is generally susceptible to β-lactam/β-lactamase inhibitor combinations. Following clinical improvement and discharge, oral amoxicillin-clavulanate was prescribed to maintain anaerobic and gram-positive coverage. Metronidazole was added as an adjunct to enhance anaerobic efficacy, given the organism’s obligate anaerobic nature and the difficulty in definitively ruling out co-infection due to the limitations of standard culture and the chronicity of the lesion. The Kingfield MetaCAP test, a targeted metagenomic capture and sequencing method, was employed due to its enhanced sensitivity for detecting low-abundance or slow-growing pathogens in clinical samples. Compared with conventional 16S rRNA sequencing, MetaCAP provides improved resolution at the species level and better detection of anaerobic bacteria, such as *Lawsonella clevelandensis*, which may be underrepresented or missed entirely by broad-spectrum or untargeted metagenomic approaches due to low biomass or poor amplification. Based on a review of the literature, she was treated with an intravenous drip of piperacillin-tazobactam (4.5 g q8h) for 16 days, during which ultrasound-guided abscess puncture and irrigation were again performed, leading to clinical improvement and her subsequent discharge. Post-discharge, she continued treatment with oral amoxicillin-clavulanate potassium (2.0 g Bid) and metronidazole (1.2 g Tid). A follow-up examination 1 month after discharge showed no signs of redness, swelling, or pain in the left breast. Ultrasound imaging revealed a reduction in the abscess size and multiple sheet-like low echoes (Fig. 2). During the telephonic follow-up on October 16, the patient reported that she had stopped taking medication a month after discharge from the hospital and had not experienced any further redness, swelling, or pain in her left breast. The medical history of the patient was unremarkable, with no prior pulmonary tuberculosis, trauma, or breast surgery. A chest computed tomography scan was normal, blood glucose levels were within normal limits, and both her complete blood count and C-reactive protein were negative.

**Table 1:** MetaCAP detection data of pus.

Genus type	Name	Series number	Relative abundance	Composite group species name	Series number	Confidence level
G**+**	Lawsonella	17,296	43.72%	*Lawsonella clevelandensis*	17,296	99%
G**+**	Finegoldia	7454	18.84%	*Finegoldia magna*	7454	99%
G**−**	Compylobacter	4660	11.78%	*Compylobacter ureolyticus*	4648	99%
G**−**	Dialister	2947	7.45%	*Dialister micraerophilus*	2945	99%
G**−**	Prewotella	1110	2.80%	*Prewotella buccalis*	1042	99%
G**−**	Mobiluncus	686	1.73%	*Mobiluncus curtisii*	624	99%
G**−**	Porphyromonas	577	1.46%	*Porphyromonas bennonis*	564	99%

MetaCAP = metagenomics capture.

To explore potential predisposing factors for recurrent abscess formation, the patient underwent an initial screening for common structural and immunological abnormalities. Fasting blood glucose was normal, effectively excluding overt diabetes mellitus. Chest computed tomography imaging revealed no evidence of pulmonary tuberculosis or systemic lesions. Her complete blood count, including leukocyte and lymphocyte profiles, showed no abnormalities suggestive of underlying immune deficiency. During the 2 hospitalizations, tests for female tumor markers (−), hepatitis B, syphilis, HIV, and hepatitis C were all negative, and there was no clinical or radiographic evidence suggestive of ductal ectasia or immunocompromise. The absence of prior surgeries, trauma, or autoimmune symptoms further reduced suspicion of structural or immunological etiology. Table 2 presents the patient’s visit schedule. Ultrasonic findings and abscess volume are documented in Table 3.

**Table 2:** Patient’s visit schedule.

Time	Symptoms	Physical signs	Relevant examinations	Interventions	Outcomes
August 2018	Stabbing pain in the upper outer quadrant of the left breast, with local skin redness and swelling	Treated outside the hospital, data temporarily missing	B-ultrasound from an external hospital indicated breast abscess formation	Ultrasound-guided abscess puncture and drainage performed at an external hospital	Improved and discharged
September 2019	Recurrence of stabbing pain in the upper outer quadrant of the left breast, local skin redness and swelling, with spontaneous skin rupture and pus discharge	Treated outside the hospital, data temporarily missing	Treated outside the hospital, data temporarily missing	Cefathiamidine was given for antiinfective treatment at an external hospital	Poor efficacy
October 2019	Stabbing pain in the upper outer quadrant of the left breast, local skin redness and swelling, rupture and pus discharge	Local skin redness and swelling with tenderness; skin surface ulcerated with bloody purulent discharge	Ultrasound in hospital: patchy hypoechoic area under the left nipple (33 × 20 × 15 mm)	Abscess incision and drainage; positive acid-fast bacilli smear; anti-TB treatment started	Improved after quadruple anti-TB treatment; switched to isoniazid monotherapy until 2024; intermittent penicillin infusions
June 2024	Left breast redness, swelling, pain, nipple pus discharge	Local skin redness, swelling, tenderness, nipple pus	Ultrasound-guided abscess aspiration (3 mL pus); FNA thyroid nodule (suggested papillary thyroid carcinoma)	Admitted; MetaCAP detected *Lawsonella clevelandensis*; empirical piperacillin-tazobactam 16 d; oral amoxicillin-clavulanate + metronidazole post-discharge	Improved; no recurrence by follow-up; reduced lesion size on ultrasound

MetaCAP = metagenomics capture.

**Table 3:** Ultrasonic findings and abscess volume.

Date	Ultrasonic report	Size (mm)	Abscess volume (mL)
October 1, 2019	Localized hypoechoic area beneath the left nipple	33 × 20 × 15	5.15
August 2, 2020	Patchy hypoechoic area adjacent to the nipple	25 × 18 × 11	2.57
March 20, 2021	Hypoechoic area under the left nipple	15 × 13 × 6	0.61
September 16, 2022	Mixed echo near the nipple	41 × 39 × 16	13.30
June 18, 2024	Mixed echo mass at 2–3 o’clock position	28 × 18 × 10	2.62
June 29, 2024	Hypoechoic area at 2–3 o’clock position	26 × 15 × 5	1.01
August 5, 2024	Multiple patchy hypoechoic areas	10 × 7 × 4; 8 × 7 × 4; 4 × 3 × 2	0.28

This case is clinically notable for the prolonged misdiagnosis and self-treatment period exceeding 5 years, during which the patient was mistakenly treated for breast tuberculosis due to the partially acid-fast nature of *Lawsonella clevelandensis*. In contrast to previously reported cases, which were promptly identified and managed, this is the first known instance of such a long-term, self-managed infection with *Lawsonella clevelandensis* in an immunocompetent host. The delayed identification via metagenomic sequencing underscores the diagnostic challenges associated with this organism and illustrates the limitations of relying solely on acid-fast staining and conventional tuberculosis markers in chronic breast infections.

## 3. Discussion and conclusions

*Lawsonella clevelandensis* is recognized as a partially acid-fast, obligately anaerobic, nonspore-forming, catalase-positive Gram-positive bacterium. In 2016, the U.S. Centers for Disease Control and Prevention categorized it under a new genus and species within the suborder Corynebacteriales.^[1]^

Initially identified in human pus samples in 2013,^[2]^ infections by this bacterium are rare, presenting predominantly as abscesses in locations, such as the breast,^[3,4]^ spine,^[5]^ abdomen,^[6,7]^ liver,^[8]^ post-vascular graft sites,^[9,10]^ and as infectious chronic abdominal aortic aneurysm ruptures.^[11]^ The bacterium affects individuals aged between 2 and 81 years, with no significant gender predilection, typically occurring in postoperative or immunocompromised patients.^[8]^

The growth of *Lawsonella clevelandensis* is notably slow, with optimal growth in anaerobic conditions and poor growth in aerobic environments. Conventional culture techniques are often ineffective for detection, with diagnosis primarily relying on gene sequencing.^[12]^ Due to its clinical presentation, which can mimic other acid-fast bacilli infections like those caused by *Nocardia* and *Mycobacterium tuberculosis*,^[7]^ there is a high risk of misdiagnosis and inappropriate treatment. Recent findings suggest that *Lawsonella clevelandensis* is a normal component of human flora, frequently colonizing oily skin areas, such as nasal creases and eyebrows, as reported by Escapa et al in 2018.^[13]^ In 2021, Polak-Witka et al also found that *Lawsonella clevelandensis* is one of the common bacteria on the scalp surface and under the hair follicle infundibulum,^[14]^ thereby indicating its role as an opportunistic pathogen.

Regarding treatment, *Lawsonella clevelandensis* is responsive to a variety of commonly used antibiotics, including β-lactams, macrolides, quinolones, cephalosporins, and rifampicin, all demonstrating low to very low minimum inhibitory concentrations.^[15]^ The prognosis for infected patients is favorable, with timely surgical intervention, such as debridement, drainage, and appropriate antibiotic therapy proving effective. Ultrasound-guided puncture and irrigation offer significant advantages over traditional surgical approaches,^[16]^ although specific guidelines for the duration of antimicrobial treatment have yet to be established.

In our case review, many infected individuals had a history of surgery or compromised immune function. The case under discussion involved a middle-aged woman with no prior health issues who experienced a sudden onset of a breast abscess, initially misdiagnosed as “breast tuberculosis.” The improvement following antituberculosis therapy suggested rifampicin sensitivity, supporting findings by Goldenberger et al.^[15]^ Despite receiving intermittent penicillin treatment, which temporarily alleviated symptoms, the patient’s irregular and incomplete treatment led to recurrent disease manifestations. During her recent hospital stay, 2 ultrasound-guided abscess puncture and flushing procedures were performed, resulting in significant symptom improvement. This case highlights the importance of accurate pathogen identification through nucleic gene sequencing to ensure correct diagnosis and treatment, distinguishing between *Lawsonella clevelandensis* infections and other similar pathogenic conditions. The empiric antimicrobial regimen was designed to broadly target anaerobic and facultative organisms based on the chronicity of infection and the possibility of polymicrobial flora. Although in vitro susceptibility testing for *Lawsonella clevelandensis* was not available in this case, prior reports have shown the organism to be susceptible to multiple β-lactams and metronidazole.^[15]^ The selected antibiotics were thus based on established susceptibility profiles and the anatomical and microbiological context, balancing empirical breadth with targeted coverage. While metagenomic next-generation sequencing (mNGS) and 16S rRNA sequencing have been widely used for pathogen identification, each has limitations in detecting rare, slow-growing, or low-abundance anaerobes. In this case, the MetaCAP platform demonstrated high specificity by capturing a near-complete microbial genome from limited clinical material, allowing for accurate species-level identification with high confidence. In contrast to 16S rRNA sequencing, which may not reliably differentiate closely related species, or mNGS, which can have limited depth in low biomass samples, the targeted enrichment strategy of MetaCAP provides a practical diagnostic advantage in complex or chronic infections where traditional methods fail. This case extends existing knowledge by illustrating that *Lawsonella clevelandensis* can mimic breast tuberculosis not only histologically, but also in clinical response to anti-tubercular agents, likely due to its susceptibility to rifampicin. Furthermore, in contrast to prior reports that focused on immunocompromised or postoperative patients, our case involves a previously healthy individual with no known risk factors, thereby broadening the clinical spectrum of susceptible populations. The prolonged disease course and self-administration of antituberculosis medication further highlight the risks of misdiagnosis and the importance of early molecular diagnostic testing to guide effective management.

The study has several limitations. Firstly, the absence of a control group makes it difficult to distinguish the effects of the treatment from natural disease progression or from other external factors. Secondly, as the bacterium grows slowly and is difficult to culture, there may have been inconsistencies or errors in diagnosis, which could affect the reliability of the case identification and subsequent analyses. Lastly, the study was retrospective and thus reliant on existing medical records and histories, which may not always be accurate or comprehensive, leading to potential bias in interpreting the course of infection and treatment outcomes.

## Author contributions

**Conceptualization:** Yuzhen Liu, Xu Yue, Guohui Zhang.

**Data curation:** Yuzhen Liu, Xu Yue, Yujun Zhou.

**Formal analysis:** Yuzhen Liu, Fang Yang, Yanling Jin.

**Investigation:** Yuzhen Liu, Xu Yue, Guohui Zhang.

**Methodology:** Yuzhen Liu, Fang Yang.

**Project administration:** Yuzhen Liu.

**Resources:** Yuzhen Liu, Yujun Zhou, Junling Wang.

**Software:** Yuzhen Liu, Guohui Zhang.

**Supervision:** Yuzhen Liu, Junling Wang, Yanling Jin, Guohui Zhang.

**Validation:** Yuzhen Liu, Junling Wang, Guohui Zhang.

**Visualization:** Yuzhen Liu, Xu Yue, Guohui Zhang.

**Writing – original draft:** Yuzhen Liu, Xu Yue, Guohui Zhang.

**Writing – review & editing:** Yuzhen Liu, Xu Yue, Yujun Zhou, Junling Wang, Fang Yang, Yanling Jin, Guohui Zhang.
